# Loss of NEDD4 contributes to RTP801 elevation and neuron toxicity: implications for Parkinson's disease

**DOI:** 10.18632/oncotarget.11020

**Published:** 2016-08-02

**Authors:** Mercè Canal, Núria Martín-Flores, Leticia Pérez-Sisqués, Joan Romaní-Aumedes, Bekir Altas, Heng-Ye Man, Hiroshi Kawabe, Jordi Alberch, Cristina Malagelada

**Affiliations:** ^1^ Department of Biomedicine, Faculty of Medicine, Universitat de Barcelona, Barcelona, Catalonia, Spain; ^2^ Department of Molecular Neurobiology, Max Planck Institute of Experimental Medicine, Göttingen, Germany; ^3^ Department of Biology, Pharmacology and Experimental Therapeutics, Boston University, Boston, Massachusetts, United States of America; ^4^ IDIBAPS-Institut d'Investigacions Biomèdiques August Pi i Sunyer, Centro de Investigación Biomédica en Red sobre Enfermedades Neurodegenerativas, Barcelona, Catalonia, Spain; ^5^ Institut de Neurociències, Universitat de Barcelona, Barcelona, Catalonia, Spain

**Keywords:** Parkinson's disease, neurodegeneration, lysosome, NEDD4, ubiquitin, Pathology Section

## Abstract

Parkinson's disease (PD) is a disorder characterized by the degeneration of certain neuronal populations in the central and peripheral nervous system. One of the hallmarks of the disease is the toxic accumulation of proteins within susceptible neurons due to major impairment in the degradation/clearance protein systems.

RTP801 is a pro-apoptotic protein that is sufficient and necessary to induce neuronal death in cellular and animal models of PD. RTP801 is also upregulated in sporadic and parkin mutant PD brains. Here, we report the role of NEDD4, an E3 ligase involved in α-synuclein degradation and PD pathogenesis, in the regulation of RTP801 protein levels and toxicity. NEDD4 polyubiquitinates RTP801 in a cell-free system and in cellular cultures, and they interact physically. NEDD4 conjugates K63-ubiquitin chains to RTP801 and targets it for degradation. NEDD4 regulates RTP801 protein levels in both cultured cells and in the brain tissue. NEDD4 levels are diminished in nigral neurons from human PD brains. Interestingly, neurotoxin 6-OHDA decreases dramatically NEDD4 protein expression but elevates RTP801 protein levels. Moreover, NEDD4 protects neuronal PC12 cells from both 6-OHDA and RTP801-induced toxicity. In primary cortical neurons, NEDD4 knockdown toxicity is mediated by RTP801 since the double knockdown of RTP801 and NEDD4 abrogates the loss of phospho Ser473-Akt and the appearance of caspase-cleaved spectrin fragments.

Thus, NEDD4 ligase regulates RTP801 and is sensitive to PD-associated oxidative stress. This suggests that NEDD4 loss of function in PD could contribute importantly into neuronal death by elevating RTP801.

## INTRODUCTION

In Parkinson's disease (PD), the mechanisms by which specific subpopulations of both central and peripheral neurons degenerate are not yet elucidated [1]. Moreover, only palliative treatments exist to ameliorate the clinical manifestations of the disease but they do not prevent neuron degeneration and death [2-4].

RTP801 is a stress-regulated protein with several functions depending on the cellular context [5]. In a variety of neuronal systems, RTP801 overexpression is sufficient to trigger cell death [5, 6]. In cellular models of PD, shRNA-mediated RTP801 knockdown is protective toward cell death induced by 6-hydroxydopamine (6-OHDA) or MPP+ [6]. Consistently, RTP801 is required for cell death triggered by MPP+/MPTP in *in vivo* model of PD [6].

RTP801 triggers neuron cell death by a sequential mechanism in which it first inactivates mechanistic target of Rapamycin (mTOR) and then, as a consequence, inhibits the neuronal survival kinase Akt, which is also a substrate of mTOR [6, 7]. In human postmortem tissue, RTP801 was found to be highly upregulated in neuromelanin (NM) positive neurons in the SNpc of both sporadic [6] and parkin mutant PD patients [8] in comparison with control non-PD brains. Also, in accordance with the mechanism proposed from our *in vitro* studies, very low levels of phospho-Akt (both Serine 473 and Threonine 308) were observed in nigral PD neurons in comparison to non-PD brains [7].

One remarkable feature of RTP801 protein is its very short half-life (2-5 min) [9, 10], suggesting that its synthesis and degradation are regulated strictly and dynamically. Our previous study demonstrated that parkin, a RING E3 ligase, ubiquitinates RTP801 and targets it for ubiquitin proteasome system (UPS) [8].

Neural precursor cell expressed, developmentally down-regulated 4 (NEDD4) is one of the most abundant ubiquitin E3 ligases in mammalian neurons [11]. NEDD4 ubiquitinates proteins, targeting them for proteasomal or lysosomal degradation [12]. In developing neurons, NEDD4 plays crucial roles in axon growth and dendrite sprouting [13, 14]. In a context of PD, NEDD4 protects neurons from alpha synuclein toxicity by ubiquitinating it and mediating its lysosomal degradation [15, 16]. Interestingly, NEDD4 staining is very strong in nigral neurons containing Lewy bodies (LB) in the human Substantia Nigra (SN) and the Locus Coeruleus (LC) from patients with LB pathologies [15]. Furthermore, NEDD4 presents a single nucleotide polymorphism (SNP) that has been associated with a major risk factor for sporadic PD in a whole genome association study (GWAS) [17].

Here, we identify NEDD4 as a novel E3 ubiquitin ligase for RTP801, controlling its homeostasis. Importantly, NEDD4 is downregulated in remaining nigral neurons from PD brains. Moreover, 6-OHDA downregulates NEDD4 in neural cultures and NEDD4 deregulation contributes to toxic elevation of RTP801 in cellular models of PD.

## RESULTS

### RTP801 is degraded by the lysosomal pathway and polyubiquitinated by NEDD4

**Figure 1:**
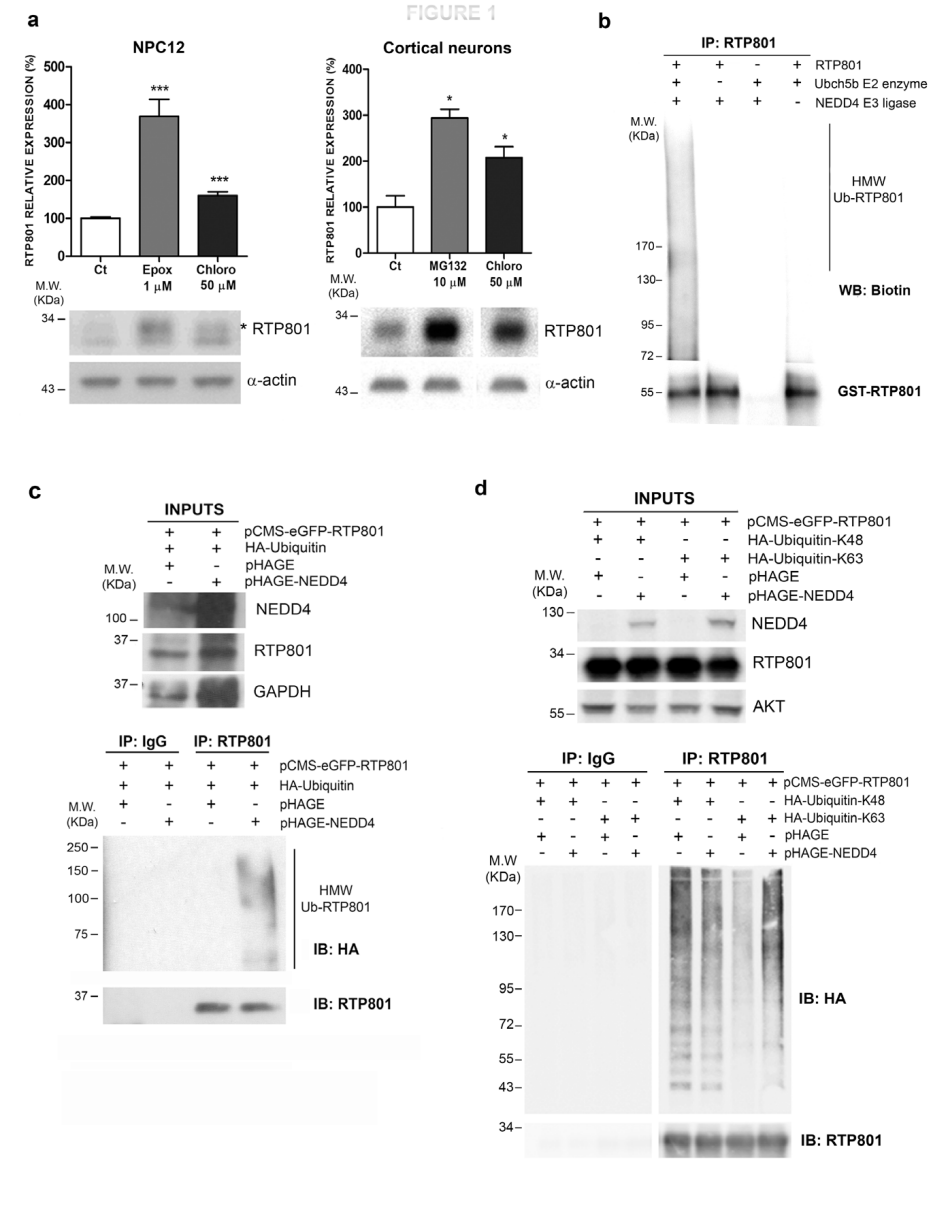
RTP801 is polyubiquitinated by NEDD4 and degraded by the lysosomal pathway **a.** **R**TP801 can be degraded by both the ubiquitin proteasome system and by the lysosomal pathway. NGF-differentiated PC12 cells or cortical neurons were treated during 4 hours with 1 μM epoxomycin, 10 μM MG132 or 50 μM chloroquine, and cell lysates were subjected to Western Blot. Membranes were probed first for RTP801 and then with α-actin, as a loading control. All samples were immunoblotted in the same membrane, but one irrelevant lane was deleted in the second panel. Graphs represent densitometric values (mean ± SEM) normalized to α-actin of three independent experiments in triplicates. Student's *t*-test, ***P < 0.001 and *P < 0.05 *versus* controls. **b.** NEDD4 polyubiquitinates RTP801 in a cell free assay. Recombinant NEDD4 E3 ligase, recombinant GST-RTP801, UbcH5b E2 enzyme, E1 enzyme, biotinylated ubiquitin and ATP were mixed and incubated at 37°C for 90 min. RTP801 was immunoprecipitated and immunocomplexes were analyzed by Western Blot. The membrane was incubated with Avidin/Biotin and then with chemiluminiscent peroxidase substrate solution (upper panel) and reprobed for RTP801 (lower panel). A representative image of three independent experiments is shown. (HMW Ub-RTP801, high molecular weight ubiquitinated RTP801) **c.**. HEK293 cells were transfected with pHAGE or pHAGE-NEDD4 along with HA-ubiquitin and pCMS-eGFP-RTP801 constructs. Forty-eight hours post-transfection either RTP801 was immunoprecipitated or non-specific rabbit immunoglobulins (Rb IgG) were added. Whole cell lysates (inputs) and the immunocomplexes were analyzed by Western Blot with anti-HA, anti-RTP801, anti-NEDD4 and anti-GAPDH (loading control) antibodies. A representative image of two independent experiments is shown. HMW Ub-RTP801 = High molecular weight ubiquitinated RTP801; IP = immunoprecipitation; IB = immunoblot. **d.** NEDD4 polyubiquitinates RTP801 with Ub-K63 chains. HEK293 cells were transfected with pCMS-eGFP-RTP801, along with pRK5-HA-Ub-K48 or pRK5-HA-Ub-K63 and pHAGE or pHAGE-NEDD4 as indicated. Forty-eight hours later, cultures were harvested and RTP801 was immunoprecipitated. Non-specific rabbit immunoglobulins (Rb IgG) were used as a negative control. Whole cell lysates (inputs) and RTP801 immunocomplexes were resolved in a Western Blot. Membrane was probed for HA, and reprobed for RTP801, for NEDD4 and for AKT as loading control. All samples were immunoblotted in the same membrane, but some irrelevant bands were deleted. Note the high molecular weight smears corresponding to polyubiquitinated RTP801. A representative image of three independent experiments is shown. IP = immunoprecipitation; IB = immunoblot.

In our previous work we showed that RTP801 has a very short half-life and is mostly degraded by the proteasome [8-10]. Hence, we first asked whether lysosomal pathway could contribute to RTP801 protein degradation. As cellular models we used NGF-differentiated PC12 cells, a cell line that resembles sympathetic neuroblasts which is a neuronal population also affected in PD [3, 18], and rat primary cortical neurons, that are also sensitive to 6-OHDA [19] or alpha-synuclein toxicity [20]. We first exposed the cultures to chloroquine, a lysosomotropic agent that prevents endosomal acidification and thus inhibits lysosomal fusion and protein degradation [21, 22]. Sister cultures were treated with proteasome inhibitors epoxomicin or MG132. Western immunoblotting (WB) showed that RTP801 was accumulated upon the inhibition of the proteasome, as previously described [8]. Interestingly, chloroquine induced a significant elevation of RTP801 after 4-hour exposure in both cultured cell types (Figure 1a).

Given that ubiquitination is involved in lysosomal degradation [23], we next investigated which E3 ligase could mediate lysosomal degradation of RTP801. We investigated NEDD4, which is an E3 ligase involved in PD by regulating alpha-synuclein proteostasis. Hence, we assessed whether recombinant NEDD4 polyubiquitinated RTP801 in a cell free assay. For this purpose, we incubated recombinant NEDD4, recombinant GST-RTP801, biotinylated Ubiquitin, an E1 ubiquitin-activating enzyme, and UbcH5b at 37°C for 90 min. After incubation, RTP801 was immunoprecipitated and analyzed by WB. By using an anti-biotin antibody, we observed the appearance of high molecular weight ubiquitinated RTP801 (HMW-Ub RTP801) species when all the enzymes and the substrates were present in the reaction (Figure 1b). Hence, NEDD4 is able to directly ubiquitinate RTP801 in a cell-free system.

To explore whether this process takes place in a cellular model, wild type (WT) NEDD4, RTP801, and Hemagglutinin (HA)-tagged ubiquitin (HA-Ub) were overexpressed in HEK293 cells. Two days later, cells were harvested and RTP801 was immunoprecipitated. WB analysis of the immunocomplexes showed that ectopic NEDD4 significantly increased the appearance of HMW-Ub RTP801 with different lengths of polyubiquitin chains (Figure 1c). These results suggest that NEDD4 enhances polyubiquitination of RTP801 in cellular cultures.

To investigate which kind of ubiquitin chains NEDD4 uses preferentially with RTP801, we transfected HEK293 cells with expression vectors for pCMS-eGFP-RTP801, NEDD4, and modified HA-Ubiquitin. The ubiquitin plasmids have all the lysine residues mutated to arginine except for 48^th^ or 63^rd^ lysine residue (K48 or K63). This preferentially leads to the formation of K48- or K63-linked polyubiquitin chains at the substrates. WB analysis of RTP801 immunoprecipitates showed that the RTP801 HMW smear was increased only in the presence of overexpressed NEDD4 along with HA-Ub-K63. Thus, NEDD4 polyubiquitinates RTP801 preferentially with K63-linked polyubiquitin chains (Figure 1d). Note that with HA-Ub-K48, the appearance of RTP801 HMW smear is independent of ectopic NEDD4. This indicates that RTP801 is ubiquitinated with K48 chains by other ligases, like parkin, and degraded by the proteasome [8].

In conclusion, RTP801 can be subjected to lysosomal degradation and is conjugated with K63-linked polyubiquitin chains by NEDD4.

### NEDD4 and RTP801 interact in cellular models

**Figure 2:**
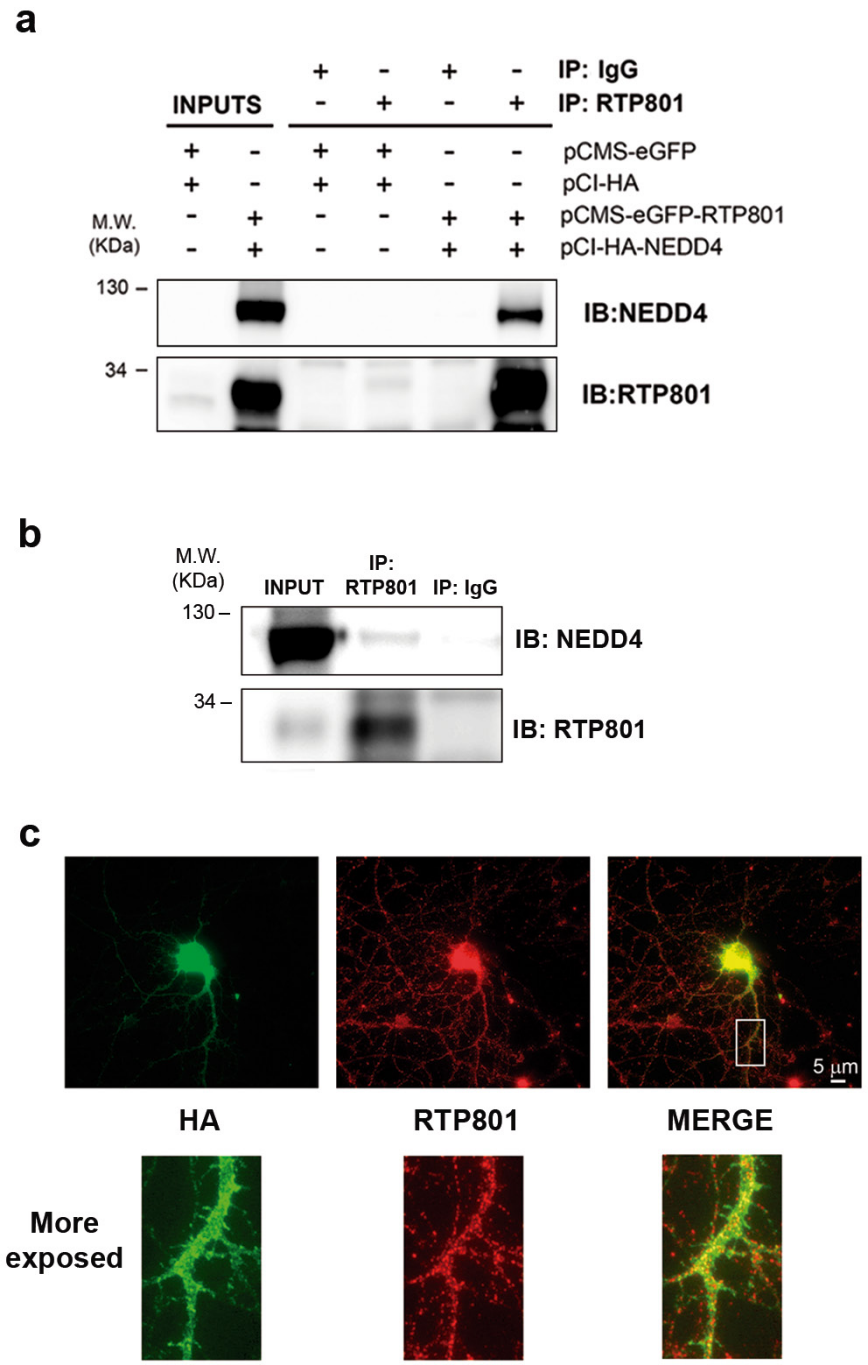
NEDD4 and RTP801 interact physically **a.** **NEDD4 co-immunoprecipitates with RTP801 in cells exposed to DSP**. HEK293 cells were co-transfected with empty vectors pCMS-eGFP and pCI-HA or with pCMS-eGFP-RTP801 and pCI-HA-NEDD4. Twenty-four hours post-transfection cells were exposed to cross-linker DSP for 2 hours at 4°C prior harvesting. RTP801 was immunoprecipitated and the samples were analyzed by Western Blotting. Membranes were probed with anti- NEDD4 and anti-RTP801 antibodies. Representative images are shown of at least three independent experiments. IP = immunoprecipitation; IB = immunoblot. **b.**. NGF-differentiated PC12 cells were treated with 1 μM epoxomicin for 2 hours. Then, cultures were exposed to DSP at 4 °C for 2 hours prior harvesting. RTP801 immunocomplexes were resolved in a Western Blotting. The membrane was incubated with anti-NEDD4 and anti-RTP801 antibodies. A representative image is shown of at least two independent assays. IP = immunoprecipitation; IB = immunoblot. **c.** NEDD4 and RTP801 co-localize in neurons. DIV 19 primary rat cortical neurons were transfected with pCI-HA-NEDD4. Forty-eight hours post-transfection, neurons were fixed and stained with anti-RTP801 (in red) and anti-HA (in green). Scale bar, 5 μm.

In order to investigate whether NEDD4 and RTP801 interact in living cells, we overexpressed both NEDD4 and RTP801 in HEK293 cells. Cultures were incubated for 2 hours with DSP, a crosslinker agent to preserve weak interactions between proteins, and then RTP801 was immunoprecipitated. WB analysis of the immunocomplexes showed that ectopic RTP801 pulled down overexpressed NEDD4 (Figure 2a).

In order to study the complex formation in non-transfected cells, we next investigated whether the endogenous NEDD4 and RTP801 interact in NGF-differentiated PC12 cells. For this purpose, cultures were treated with 1 μM epoxomycin for 2 hours to abrogate RTP801 fast degradation and then, they were exposed to the crosslinker DSP for two hours more. Then, endogenous RTP801 was immunoprecipitated. WB analysis showed that NEDD4 was able to interact with endogenous RTP801 (Figure 2b).

We next explored whether NEDD4 and RTP801 colocalize in cultured primary neurons. We overexpressed HA-tagged NEDD4 in primary rat cortical cultures and we detected ectopic NEDD4 with an antibody against the HA epitope. Interestingly, endogenous RTP801 co-localized with HA-tagged NEDD4 in the soma and in the neurites (Figure 2c) of transfected neurons.

Taken together, these experiments suggest the physical interaction of NEDD4 and RTP801 in several cellular models.

### NEDD4 regulates RTP801 protein levels in cellular models

**Figure 3:**
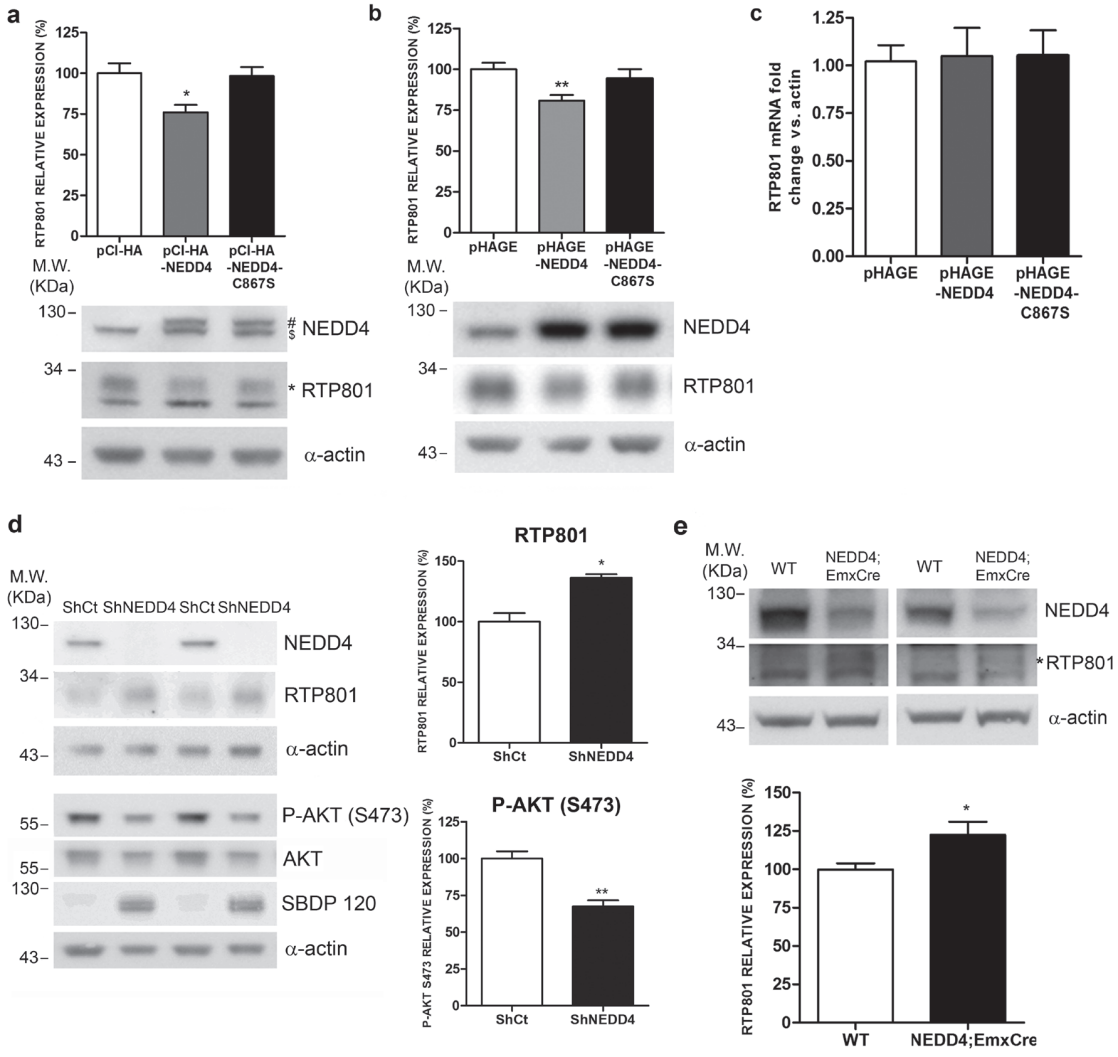
Ectopic NEDD4 regulates RTP801 in cultured cells **a.** **Ectopic NEDD4 decreases RTP801 protein levels in neuronal PC12 cells.** NGF-differentiated PC12 cells were transfected with pCI-HA, pCI-HA-NEDD4 or pCI-HA-NEDD4-C867S constructs. Forty-eight hours post-transfection cultures were harvested and analyzed by Western Blot with anti-NEDD4, anti-RTP801 and anti-α-actin antibodies. Representative immunoblots are shown along with densitometric quantification from at least three independent experiments. ($ endogenous NEDD4, # ectopic NEDD4, * specific band for RTP801). One-way ANOVA with Bonferroni multiple comparison test, *P < 0.05 *versus* pCI-HA. **b.**. DIV 8 rat primary cortical neurons were infected with lentiviruses containing the empty vector pHAGE, pHAGE-NEDD4 or pHAGE-NEDD4-C867S inactive mutant. Cell lysates were harvested 4 days later and analyzed by Western Blot with antibodies against RTP801, NEDD4 and α-actin as loading control. The graph represents RTP801 densitometries of at least three independent experiments done in triplicate. One-way ANOVA with Bonferroni multiple comparison test, **P < 0.01 *versus* pHAGE. **c.** Ectopic NEDD4 does not affect RTP801 mRNA levels. DIV 8 cortical neurons were infected with lentiviruses containing the constructs pHAGE, pHAGE-NEDD4 or pHAGE-NEDD4-C867S. RNA was extracted 4 days later, and reverse transcription-qPCR was performed to quantify RTP801 transcripts. Results are displayed as RTP801 mRNA fold change respect to α-actin mRNA levels. The graph shows values (mean ± SEM) of three independent experiments. **d.** NEDD4 knockdown increases RTP801 protein levels and is detrimental for neurons. DIV 4 cortical neurons were infected with lentiviruses containing a scrambled shRNA (ShCt) or a mix of three shRNA sequences against NEDD4 (ShNEDD4). Six days later, cells were harvested and analyzed by Western Blot. Membranes were incubated with NEDD4, RTP801, P-AKT (S473), AKT and α-spectrin antibodies. The antibody against α-actin was used as loading control. For Western blotting using the α-spectrin antibody the caspase-cleaved fragment (spectrin breakdown product 120, SBDP120) is shown. Representative immunoblots are shown along with RTP801 and P-AKT (S473) densitometries (mean ± SEM) of at least three independent experiments. Student's *t*-test, *P < 0.05 and **P < 0.01 *versus* ShCt. **e.** *NEDD4f/f*;Emx1Cre conditional knockout mice have elevated RTP801 protein levels in the cortex. Cortical lysates of 6-week old mice were analyzed by Western Blotting with anti-NEDD4 and anti-RTP801 antibodies, and then reprobed with anti-α-actin antibody as loading control. Representative immunoblots are shown along with RTP801 densitometries (mean ± SEM) of at least three independent gels. All samples were immunoblotted in the same membrane, but some irrelevant lanes were deleted. (* Specific band for RTP801) Student's *t*-test, *P < 0.05 *versus* WT.

Our next question was whether NEDD4, by polyubiquitinating RTP801, could regulate RTP801 protein levels. In neuronal PC12 cells, we overexpressed WT NEDD4 and its inactive mutant, with the catalytic cysteine 867 mutated to a serine (NEDD4-C867S). After 48 hours, cell lysates were analyzed by WB. Ectopic NEDD4 diminished in a 25-30% the levels of endogenous RTP801 protein. Interestingly, the inactive mutant form of NEDD4 did not affect RTP801 levels (Figure 3a). We obtained the same results in rat primary cortical neurons (Figure 3b).

Still in cortical neurons, either ectopic NEDD4 or its mutant did not alter the messenger RNA levels of RTP801 (Figure 3c). These results indicate that NEDD4 does not regulate the transcription of RTP801.

We next asked whether NEDD4 knockdown elevates RTP801 protein levels. Therefore, in rat primary cortical neurons, NEDD4 expression was abrogated with specific shRNAs packed in lentiviruses. Six days later, cell lysates were analyzed by WB. Upon expression of shRNA for NEDD4, almost all NEDD4 protein was knocked down as shown in the top panel of Figure 3d. We observed a significant increase of RTP801 protein levels in the absence of NEDD4. In the same conditions, we also detected that the levels of phosphorylated residue S473 of the survival kinase Akt were diminished and the levels of caspase-cleaved spectrin fragment SBDP120, as readout of toxicity, were significantly elevated (Figure 3d). Thus, NEDD4 loss induces an elevation of RTP801 and is deleterious for cultured neurons.

Then, we assessed whether the NEDD4 deletion could affect the accumulation of RTP801 in an animal model. NEDD4 knockout mice are not viable after birth. For this reason we used *NEDD4f/f;Emx1Cre* conditional knockout mice that only abolishes the expression of NEDD4 in glutamatergic neurons and glial cells in the cortex [13, 24, 25]. By WB, cortical lysates from 6-week old mice showed that the downregulation of NEDD4 was around 50%. Accordingly, RTP801 levels were increased by a 25% in the conditional mice in comparison to the control NEDD4^f/f^ littermates (Figure 3e).

Together, these results show that NEDD4 regulates protein levels of RTP801 in cultured cortical neurons and *in vivo*.

### NEDD4 protects neuronal PC12 cells from ectopic RTP801 toxicity

**Figure 4:**
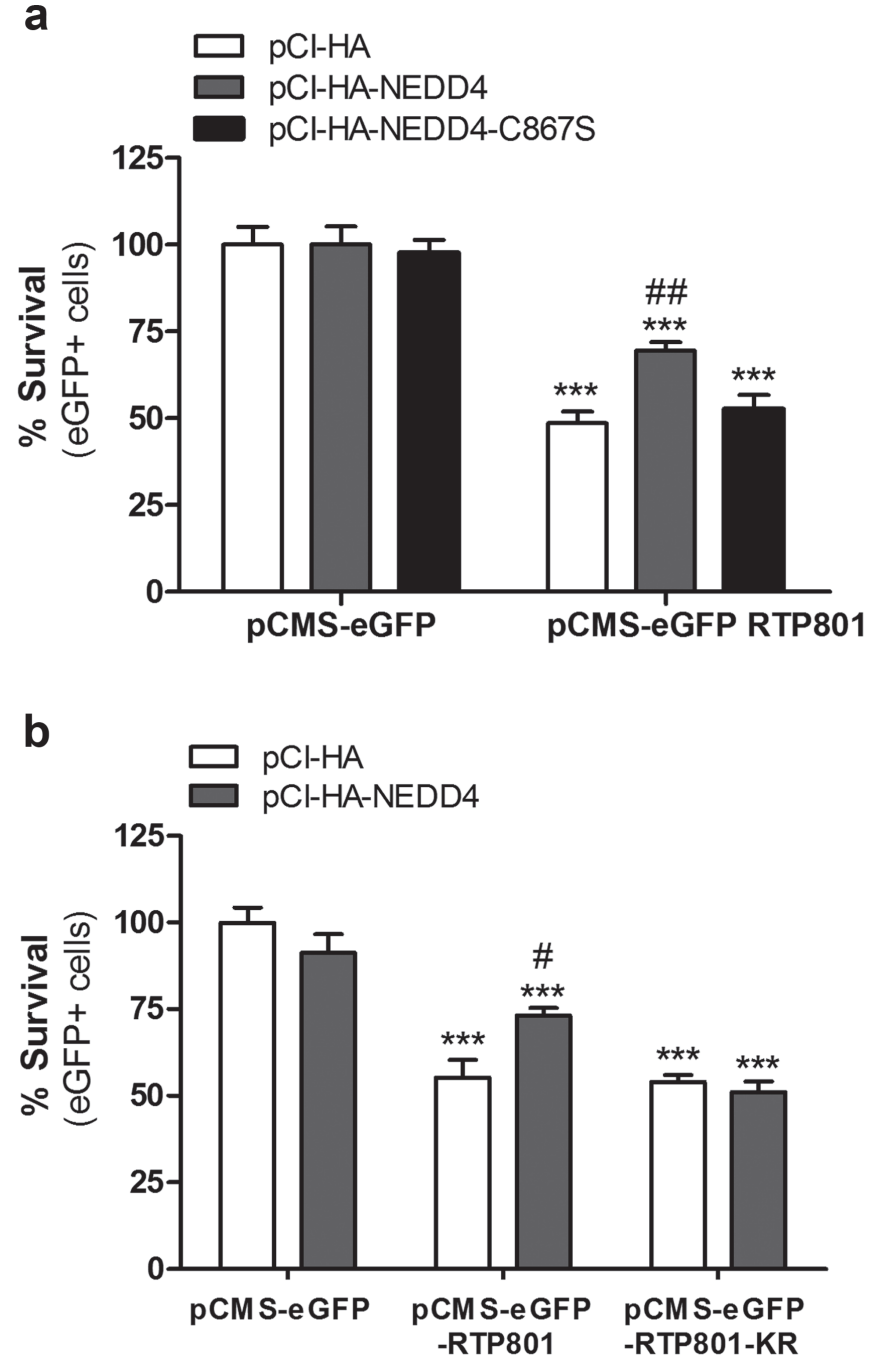
NEDD4 partially prevents from RTP801-induced cell death **a.** **Ectopic WT NEDD4 protects from RTP801-induced cell death**. NGF-differentiated PC12 cells were co-transfected with pCI-HA, pCI-HA-NEDD4 or pCI-HA-NEDD4-C867S constructs, together with either pCMS-eGFP or pCMS-eGFP-RTP801. Cells were fixed 24 hours later and cell survival (eGFP+ cells) scored under fluorescence microscopy. The graph represents mean ± SEM of at least three independent experiments in quadruplicates. One-way ANOVA with Bonferroni multiple comparison test, ***P < 0.001 *versus* pCI-HA/pCMS-eGFP, ^##^*P* < 0.01 *versus* pCI-HA/pCMS-eGFP-RTP801. **b.**. NGF-differentiated PC12 cells were co-transfected with pCI-HA or pCI-HA-NEDD4 together with pCMS-eGFP, pCMS-eGFP-RTP801 or pCMS-eGFP-RTP801-KR. Twenty-four hours later, cells were fixed and eGFP+ surviving cells scored using fluorescence microscopy. The graph represents mean ± SEM of at least three independent experiments in quadruplicates. One-way ANOVA with Bonferroni multiple comparison test, ***P < 0.001 *versus* pCI-HA/pCMS-eGFP and ^#^*P* < 0.05 *versus* pCI-HA/pCMS-eGFP-RTP801.

Overexpression of RTP801 induces around 50% of cell death in cultured neurons and non-proliferating NGF-differentiated PC12 cells [6, 8]. With this rationale, we overexpressed pCMS-eGFP empty vector or with pCMS-eGFP-RTP801 along with WT NEDD4 and the inactive mutant NEDD4-C867S. Twenty-four hours later, cell viability was assessed by scoring the number of living eGFP+ cells under epifluorescence microscopy. Only WT NEDD4 but not NEDD4-C867S protected cells from RTP801 toxicity (Figure 4a). Indeed, NEDD4 did not exert protection over non-ubiquitinable RTP801-KR mutant, with the 6 lysines mutated to arginines. These results support our notion that NEDD4 protects from RTP801 toxicity by ubiquitinating it (Figure 4b).

### PD neurotoxin 6-OHDA decreases NEDD4 and elevates RTP801 protein levels

**Figure 5:**
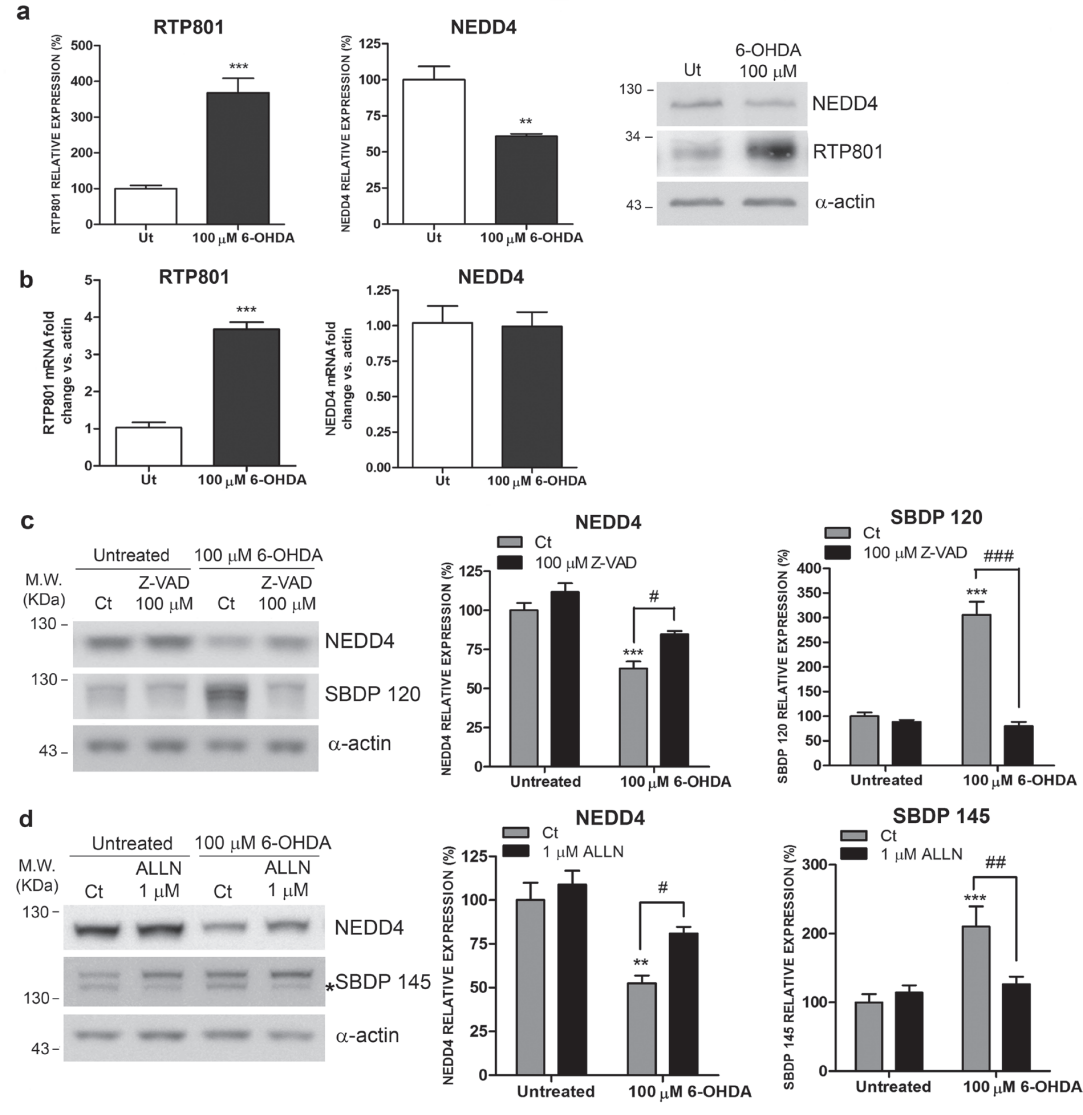
NEDD4 is decreased in a 6-OHDA PD cellular model **a.** **NEDD4 protein levels are diminished in 6-OHDA-treated neuronal PC12 cells.** NGF-differentiated PC12 cells were exposed to 100 μM 6-OHDA for 16 hours prior harvesting. Cell lysates were analyzed by Western Blot with antibodies against NEDD4, RTP801, as well as α-actin antibody as loading control. Representative immunoblots are shown along with densitometries represented as mean ± SEM of at least three independent experiments. Student's *t*-test, **P < 0.01 and ***P < 0.001 *versus* Ut (Untreated). **b.** NEDD4 mRNA levels are not modified in 6-OHDA-treated neuronal PC12 cells. NGF-differentiated PC12 cells were exposed to 100 μM 6-OHDA for 8 hours. RNA was extracted and reverse transcription-qPCR was performed. The graphs show values (mean ± SEM) of three independent experiments. Student's *t*-test, ***P < 0.001 *versus* ut (untreated). **c.** NEDD4 is cleaved by caspases after 6-OHDA exposure. Neuronal PC12 cells were treated with 100 μM Z-VAD-FMK pan-caspase inhibitor for 1 hour prior to 6-OHDA exposure. Sixteen hours later, cultures were harvested and analyzed by Western Blot. Membranes were incubated with antibodies against NEDD4 and α-spectrin as well as an α-actin antibody as loading control. Graphs represent mean ± SEM of NEDD4 and caspase-cleaved α-spectrin fragment (spectrin breakdown product 120 KDa, SBDP 120) densitometric quantification of at least three independent experiments. One-way ANOVA with Bonferroni multiple comparison test, ***P < 0.001 *versus* Untreated/Ct and ^#^*P* < 0.05, ^###^*P* < 0.001 *versus* 100 μM 6-OHDA/Ct. **d.** NEDD4 is cleaved by calpains after 6-OHDA exposure. Neuronal PC12 cells were treated with 1 μM ALLN calpain inhibitor for 1 hour prior to 6-OHDA exposure. Sixteen hours later, cultures were harvested and subjected to Western Blot. Membranes were incubated with anti-NEDD4 and anti-α-spectrin antibodies and with anti-α-actin as loading control. Graphs represent mean ± SEM of NEDD4 and calpain-cleaved α-spectrin fragment (spectrin breakdown product 145 KDa, SBDP 145) densitometric quantification of at least three independent experiments. One-way ANOVA with Newman-Keuls multiple comparison test, **P < 0.01, ***P < 0.001 *versus* Untreated/Ct and ^#^*P* < 0.05, ^###^*P* < 0.001 *versus* 100 μM 6-OHDA/Ct.

In our previous works we described that 6-OHDA elevated RTP801 protein levels transcriptionally and also posttranslationally, by interfering in its proteasomal degradation [6, 8, 10]. We observed that in culture, 6-OHDA was deleterious for parkin E3 ligase activity/levels [8]. Based on this background, we next asked whether 6-OHDA also influences the levels of NEDD4 in cellular cultures. In NGF-differentiated PC12 cells, 6-OHDA diminished NEDD4 protein levels but did not affect the levels of its mRNA. On the contrary, 6-OHDA elevated both mRNA and protein levels of RTP801 as reported previously (Figures 5a and 5b) [6, 8].

In order to investigate whether proteases such as caspases and calpains could be responsible for NEDD4 decrease, we pre-treated NGF-differentiated PC12 cells for one hour with 100μM of Z-VAD-FMK, a pan caspase inhibitor, or with 1μM of ALLN, a calpain inhibitor, and then, they were exposed to 6-OHDA. Both caspase and calpain independent inhibition partially prevented the loss of NEDD4 induced by 6-OHDA. The high levels of caspase-cleaved (SBDP120) and calpain cleaved-spectrin fragments (SBDP145) confirmed the toxicity of 6-OHDA (Figures 5c and 5d).

Taken together, these results indicate that caspase and calpain activation contribute to the loss of NEDD4 protein levels induced by 6-OHDA.

### Ectopic NEDD4 protects from 6-OHDA and prevents RTP801 elevation

**Figure 6:**
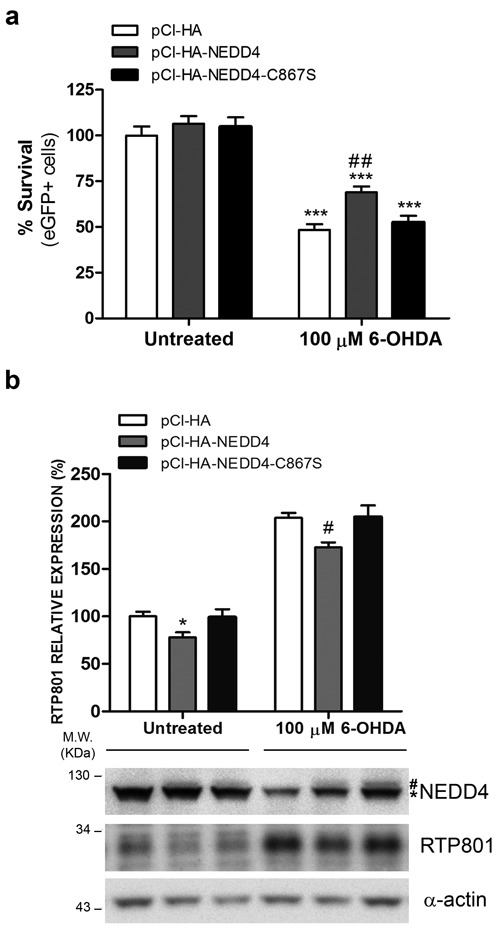
NEDD4 protects from 6-OHDA-induced cell death and decreases RTP801 elevation **a.** Ectopic NEDD4 WT partially prevents from 6-OHDA-induced cell death. NGF-differentiated PC12 cells were co-transfected with pCI-HA/pCMS-eGFP, pCI-HA-NEDD4/pCMS-eGFP or pCI-HA-NEDD4-C867S/pCMS-eGFP vectors with a 4:1 ratio. Thirty-two hours later, cell cultures were exposed to 100 μM 6-OHDA for 16 hours. Then, eGFP+ surviving cells were scored under fluorescence microscopy. The graph shows mean ± SEM of at least three independent experiments done in quadruplicate. One-way ANOVA with Bonferroni multiple comparison test, ***P < 0.001 *versus* Untreated/pCI-HA and ^##^*P* < 0.01 *versus* 100 μM 6-OHDA /pCI-HA. **b.** Ectopic NEDD4 reduces RTP801 elevation after 6-OHDA exposure. NGF-differentiated PC12 cells were transfected with pCI-HA, pCI-HA-NEDD4 or pCI-HA-NEDD4-C867S. Thrity-two hours later, cell cultures were exposed to 100 μM 6-OHDA for 16 hours and cell lysates were analyzed by Western Blot. Membranes were incubated with antibodies against NEDD4, RTP801 and α-actin as loading control (* endogenous NEDD4, # ectopic NEDD4). Low signal of ectopic NEDD4 maybe due low sensitivity of NEDD4 antibody towards the human NEDD4 protein, as compared to endogenous rat NEDD4. Representative immunoblots are shown along with RTP801 densitometric normalized quantification (mean ± SEM) from at least three independent experiments. One-way ANOVA with Bonferroni multiple comparison test, *P < 0.05 *versus* Untreated/pCI-HA, ^#^*P* < 0.05 *versus* 100 μM 6-OHDA/pCI-HA.

Since 6-OHDA decreased NEDD4 protein levels and this could be related with the elevation of RTP801, we first asked whether NEDD4 restitution could be protective against this neurotoxin. In fact, we chose to use the 6-OHDA model instead of over expressing alpha-synuclein because NEDD4 degrades alpha synuclein [15]. Thus, we overexpressed wild type or the inactive mutant of NEDD4 in NGF-differentiated PC12 cells and then exposed the cultures to 6-OHDA. By assessing cell survival, based on the number of eGFP+ cells with non-condensed or non-pyknotic nuclei (Hoechst staining), we concluded that ectopic WT NEDD4 partially prevented from 6-OHDA-induced cell death while the overexpression of the mutant had almost no effect (Figure 6a).

We next investigated whether the protection conferred by NEDD4 could be related with the modulation of RTP801 protein levels. WT NEDD4 or its inactive mutant NEDD4-C867S were overexpressed in NGF-differentiated PC12 cells. After 32 hours, the cultures were exposed to 6-OHDA and cell lysates were analyzed by WB (Figure 6b). We observed a significant effect over RTP801 levels. Ectopic NEDD4 partially abrogated the 6-OHDA-induced levels of RTP801, in comparison to control or the NEDD4 inactive mutant.

Taken together, we conclude that NEDD4 protects from 6-OHDA toxicity and downregulates RTP801 induced by the toxin.

### NEDD4 loss is toxic due to RTP801 elevation in cortical neurons

**Figure 7:**
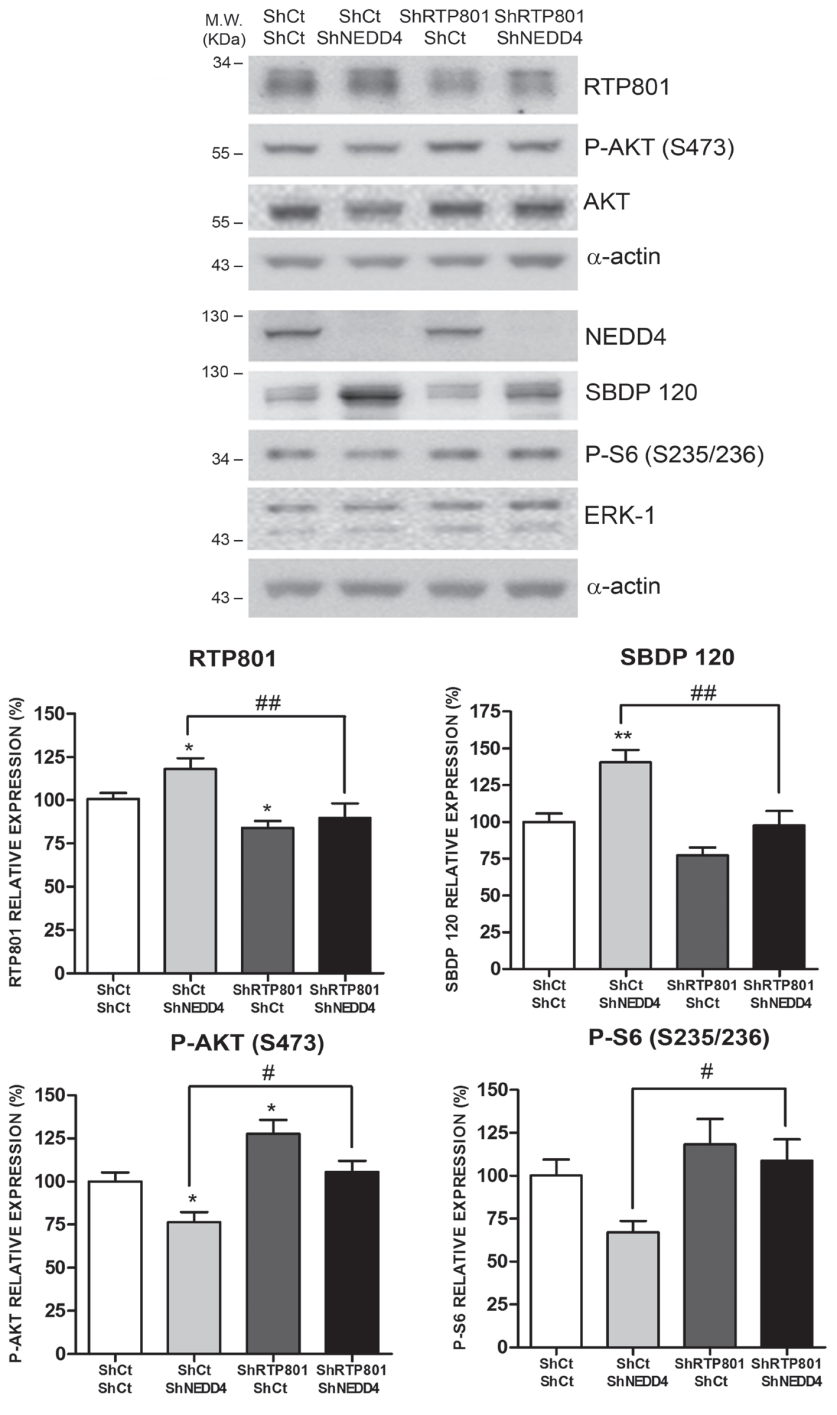
NEDD4 knockdown toxicity is dependent on RTP801 protein DIV 5 primary rat cortical neurons were infected with lentiviruses containing a scrambled shRNA (ShCt) or a shRNA against RTP801 (ShRTP801). Two days later, neurons were transduced with a mix of three shRNA sequences against NEDD4 (ShNEDD4) or the corresponding control shRNA (ShCt). Cell lysates were analyzed 4 days later by Western Blot. Membranes were incubated with RTP801, NEDD4, P-AKT (S473), AKT, P-S6 (S235/236), ERK1/2 and α-spectrin antibodies, and with α-actin antibody as loading control. For α-spectrin the caspase-cleaved fragment (spectrin breakdown product 120, SBDP120) is shown. Representative immunoblots are shown along with densitometries (mean ± SEM) of at least two independent experiments done in triplicate. One-way ANOVA with Newman-Keuls multiple comparison test, *P < 0.05, **P < 0.01 *versus* ShCt/ShCt and ^#^*P* < 0.05, ^##^*P* < 0.01 *versus* ShCt/ShNEDD4.

We next investigated whether the toxicity of NEDD4 knockdown is mediated by RTP801 elevation in neurons. Rat primary cortical neurons were sequentially infected with lentiviral particles to knockdown RTP801 first and then NEDD4. WB analysis of the cell lysates showed that knocking down NEDD4 elevated RTP801 along with caspase-cleaved spectrin fragment SBDP120. Levels of phospho-S473-Akt and phospho-S6, as mTORC2 and mTORC1 activity readout respectively, were both reduced, although only P-S473-Akt was statistically significant. Indeed, knocking down RTP801 alone elevated the phosphorylation of Akt. Importantly, knocking down both RTP801 and NEDD4 abrogated RTP801 elevation and prevented the decrease of phospho-S473-Akt and phospho-S235/236-S6 (Figure 7).

These results conclude that NEDD4 has a robust functional relationship with RTP801 in the regulation of downstream targets mTOR and Akt.

### NEDD4 levels are diminished in nigral neurons in PD brains

**Figure 8:**
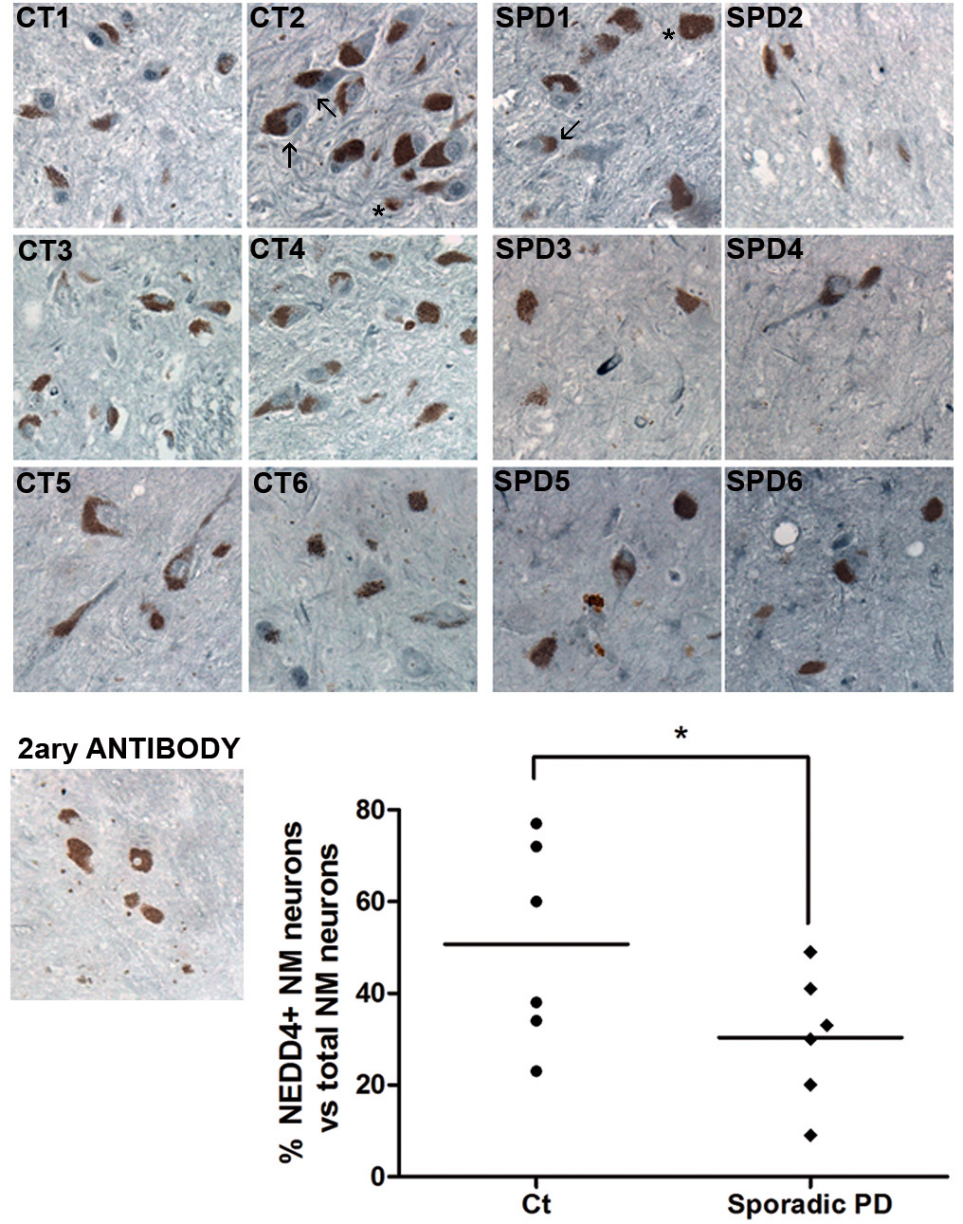
NEDD4 is decreased in pigmented nigral neurons from sporadic PD patients SNpc human postmortem sections from control individuals (CT1-CT6) and sporadic PD patients (SPD1-SPD6) were immunostained for NEDD4 (grey-blue). Note the presence of neuromelanin (NM) granules (brown) within the somas of dopaminergic neurons. Neuromelanin-positive (NM+) neurons were scored positive for NEDD4 when detectable staining was observed. For each case, the percentage of NM+ and NEDD4+ neurons versus total NM+ neurons was represented in the graph. A control section was immunostained with no primary antibody as negative control. Arrows indicate some of the positive cells stained for NEDD4 and asterisks show some of the negative cells for NEDD4. Counting was performed in blind conditions. Student's *t*-test, *P < 0.05.

Previous studies showed that NEDD4 was upregulated in human nigral neurons containing Lewy Bodies (LBs) from patients diagnosed with Lewy bodies disease (LBD) [15]. However, only 15% of nigral neurons contains alpha synuclein-reactive LBs [26]. For this reason, we extended the study to all nigral neurons in SNpc sections from sporadic PD patients. We immunostained 6 sporadic PD and 6 control cases with a specific antibody for NEDD4. We scored the number of nigral neurons, (visualized in brown due to neuromelanin pigment) that were positive for NEDD4 (in blue) *versus* total nigral neurons, in each case (Figure 8). We observed, independently of the presence of LBs, that the percentage of remaining nigral neurons stained for NEDD4 was lower in PD cases than in the age-matched controls (Figure 8). We did not observe staining in sections incubated only with the secondary antibody. This loss of NEDD4 expression in PD correlates well with the previously reported RTP801 elevation in nigral neurons [6, 8].

Taken together, these results show that NEDD4 is decreased in nigral neurons from PD patients that could well explain the previously reported RTP801 elevation in the same neuronal population [6, 8].

## DISCUSSION

Here, we show that RTP801, a pro-apoptotic protein involved in neuron death in cellular and animal models of PD, is a novel substrate of NEDD4. NEDD4 polyubiquitinates RTP801 by conjugating K63 ubiquitin chains. This type of posttranslational modification is associated with the lysosomal pathway. NEDD4 and RTP801 interact physically in HEK293 and in NGF-differentiated PC12 cells. NEDD4 also regulates RTP801 protein levels in both cultured cells and in a conditional NEDD4 knockout mouse model. Moreover, NEDD4 protein levels are diminished in remaining nigral neurons in human sporadic PD brains. Furthermore, we provide evidence that NEDD4 is protective against ectopic RTP801 toxicity by ubiquitinating it. We found that 6-OHDA neurotoxin exposure decreased dramatically NEDD4 protein levels and its restoration abrogated 6-OHDA-induced cell death. Ectopic NEDD4 also prevented RTP801 protein elevation induced by 6-OHDA. Finally, NEDD4 loss of function toxicity was mediated by RTP801 by inactivating mTOR/Akt kinases.

The finding that RTP801 is also degraded by the lysosomal system in neurons was yet not known. In lymphocytes treated with dexamethasone, RTP801/REDD1 mediated the fusion of lysosomes with autophagosomes, and therefore it mediated autophagy [27]. Interestingly, mTORC1 translocates to the lysosomes surface *via* the Rag-ragulator complex to be activated [28]. One can speculate that NEDD4 targeting RTP801 to the lysosomal degradation pathway is necessary for lysosomal mTOR translocation/activation; therefore an elevation of RTP801 could be deleterious for this process.

Our previous studies confirmed that RTP801 was mostly degraded by the proteasome. This is in line with the evidence relating proteins with short half-lives more prone to be degraded by the proteasome. However, here we found a pool of RTP801 that is degraded by the lysosomal pathway. Both proteasome and lysosome systems seem to be impaired in PD [29, 30]. Thus, based on our previous and current results, the deregulation of both processes could be contributing importantly to RTP801 elevation in the pathogenesis of PD.

We show that NEDD4 ubiquitinates RTP801, preferentially with K63-ubiquitin chains. K63-ubiquitin chains have a low affinity for the 26S proteasome subunit and they rather bind other soluble proteins, such as ESCRT-0 (Endosomal Sorting Complex Required for Transport) and its components, STAM and Hrs that block the interaction with S26 proteasomal subunit [31]. Therefore they are targeted to the lysosomal pathway. On the contrary, K48-ubiquitin chains have more affinity for Rad23 proteins, especially hHR23B, that promote proteasome binding [31].

NEDD4 binds proteins with proline rich motifs [32] or phospho-serines or phospho-threonines [33] *via* its WW binding domains. Indeed, RTP801 protein sequence has a proline rich region in the fragment close to the N-terminus that contains several serines and threonines. Interestingly, threonine residues 23 and 25 fit the consensus sequence for GSK3b kinase and could be therefore, phosphorylated [34].

To date only three E3 ligases have been described to regulate RTP801: CUL4A- DDB1-ROC1-b-TRCP E3 ligase complex, HUWE-1 and parkin. CUL4A-DDB polyubiquitinates RTP801 and mediates its proteasomal degradation in a GSK3 phosphorylation dependent manner [34]. However, other authors could not confirm these results by using a chemical inhibitor of all cullin E3 ligases [35].

We recently found that parkin polyubiquitinates RTP801 and mediates its proteasomal degradation [8]. Parkin dysfunction, due to mutations or oxidative/nitrosative stress has been consistently linked to PD [36-40]. Regarding NEDD4 involvement in PD, Tofaris and colleagues (2011) [15] first described the possible role of NEDD4 in the endosomal-lysosomal degradation of alpha-synuclein in cellular models and in yeast. However in their work, whether NEDD4 activity was altered in PD, as it is the case with parkin, was not clear.

NEDD4 knockdown elevated RTP801 and caspase-cleaved spectrin fragment SBDP120. Interestingly it also inactivated Akt by decreasing the levels of phosphorylation at the Serine-473 residue. All these events suggest that NEDD4 loss of function was toxic for neurons.

These signaling events were clearly observed in mature rat cortical neurons although they were not significant in the 6-week-old mice cortical lysates from the *NEDD4f/f;Emx1Cre* conditional knock out mice. These results suggest that in these mice, neuronal death could be masked due to compensatory mechanisms.

NEDD4 loss is toxic in cortical neurons and at least, it involves RTP801 elevation and inactivation of the mTOR/Akt kinases. In fact, knocking down RTP801 at the same time as NEDD4 prevented the loss of phospho-Akt and phospho-S6 and the appearance of caspase-cleaved spectrin fragments. NEDD4 has been related with Akt survival signaling. NEDD4 polyubiquitinates phospho-Akt at the plasma membrane to modulate Akt subcellular localization [41]; it binds to GRB10 to regulate IGF-1 and insulin signaling, including IGF1 receptor ubiquitination [42]. Interestingly, Grb10 is an mTORC1 substrate [43].

Hence, NEDD4 activity over RTP801, which in turn is a negative regulator of Akt and mTOR, would be crucial to prevent neurodegeneration.

Consistent with the toxicity induced by the loss of NEDD4 in cultured cells, we found in human PD brains that the percentage of NEDD4-stained nigral neurons were lower in PD cases than in age-matched controls. This result correlates with the previously reported elevation of RTP801 in nigral neurons in sporadic PD cases [6, 8]. However, Tofaris et al. (2011) [15] described that NEDD4 was strongly expressed in pigmented neurons from both LC and SN containing LBs, as a neuroprotectective mechanism against neurodegeneration [15]. According to literature, only 15% of the remaining nigral neurons contains LB [26]. The fact that we considered all the remaining nigral neurons could explain the differences between these two works. Whether RTP801 is elevated in LC has not been yet explored.

In conclusion, here we show that the HECT E3 ligase NEDD4 mediates the ubiquitination of proapoptotic protein RTP801 and its lysosomal degradation. We observed that NEDD4 knockdown is toxic for neurons and this toxicity is counteracted when RTP801 elevation is abrogated. Moreover, the levels of NEDD4 in the remaining nigral neurons in human PD brains are diminished. These nigral neurons also present elevated levels of RTP801. Thus, the loss of NEDD4 in PD could be leading to neurodegeneration at least in part, by contributing to RTP801 toxic accumulation.

## MATERIALS AND METHODS

### Antibodies, plasmids and materials

Rabbit polyclonal antibody against RTP801 was purchased from Proteintech Group Inc (Chicago, IL, USA). Rabbit polyclonal anti-NEDD4 (used for WB), rabbit polyclonal anti-ERK1/2 and mouse monoclonal anti-GFP antibodies were obtained from Santa Cruz Biotechnology (Dallas, TX, USA). Anti-HA antibody (used for WB), anti-P-Akt (S473), anti-P-S6 (S235/236) and anti-Akt rabbit polyclonal antibody were purchased from Cell Signaling Technology (Danvers, MA, USA). Mouse monoclonal antibody against HA-tag (used for immunofluorescence) was obtained from Sigma-Aldrich (Sant Louis, MO, USA). Ubiquitin (FK2) antibody was purchased from Enzo Life Sciences (Farmingdale, NY, USA). Rabbit polyclonal antibody against NEDD4 and GAPDH (used for immunohistochemistry) were from Abcam (Cambridge, UK). The antibody against αII-spectrin was purchased from Merck Millipore (Billerica, MA, USA). Anti-α-actin antibody was obtained from MP Biomedicals (Santa Ana, CA, USA). Horseradish peroxidase-conjugated goat anti-mouse and anti-rabbit secondary antibodies were obtained from Pierce Thermo Fisher Scientific (Rockford, IL, USA). Goat anti-mouse and anti-rabbit secondary antibodies conjugated to Alexa 488 or Alexa 568 were purchased from Thermo Fisher Scientific (Waltham, MA, USA).

pCMS-eGFP-RTP801 and pCMS-eGFP-RTP801 KR constructs were generated as previously described [6, 8]. pCI-HA-NEDD4 and pcDNA3 HA-ubiquitin were purchased from Addgene (Cambridge, MA, USA). The construct pCI-HA was a kind gift from Dr. Joan Massagué (Memorial Sloan Kettering Cancer Center, New York, NY, USA). pHAGE and pHAGE-NEDD4 constructs were kindly provided by Dr.Heng-Ye Man (Boston University, MA, USA). pRK5-HA-ubiquitin-K48 and pRK5-HA-ubiquitin-K63 were a kind gift from Dr. Bernat Crosas (Molecular Biology Institute of Barcelona, CSIC, Spain). The lentivirus packaging plasmids pMDLg/pRRE, pRSV-Rev and pMD2.G used to knockdown RTP801 were obtained from Addgene. The lentivirus packaging plasmids pHDM-Tat1b, pRC/CMV-Rev1b, pHDM-Hgpm2 and pHDM-G used to overexpress NEDD4 were kindly provided by Dr. Heng-Ye Man. All vectors were validated by DNA sequencing.

Epoxomicin, MG132, cycloheximide, Z-VAD-FMK, and ALLN were purchased from Calbiochem Merck Millipore (Billerica, MA, USA), chloroquine and CHAPS were obtained from Sigma-Aldrich, and 6-OHDA was purchased from Tocris Bioscience (Bristol, UK).

### Directed mutagenesis

pCI-HA-NEDD4-C867S and pHAGE-NEDD4-C867S constructs were obtained by mutating the catalytic cysteine (C) 867 to serine (S) of pCI-HA-NEDD4 and pHAGE-NEDD4 vectors. The QuickChange Lightning II multi site-directed mutagenesis kit (Agilent Technologies, Santa Clara, CA, USA) was used with the following primer: 5′-GTC CAGGCGATTAAAACTGGTATGAGCTCTTGGCA-3′. All new constructs were validated by DNA sequencing.

### ShRNA production

Scrambled control ShRNA (ShCt) and ShRTP801 were generated as previously described [6], based on the following sequences: ShCt, 5′- GTGCGTTGCTAGTACCAAC-3′, ShRTP801, 5′-AAGACTCCTCATACCTGGATG-3′ (specific for human, rat and mouse RTP801). The two ShRNA were inserted into the lentiviral vector pLL3.7 (Addgene).

### *NEDD4f/f;Emx1Cre* mice

In order to generate the forebrain specific *NEDD4* conditional knockout mouse, *Nedd4*^f/f^ mice [13] were crossed with *Emx1*Cre mouse [24]. *Emx1*Cre mice express Cre recombinase in the radial glia cells, resulting in flox-recombination of floxed target genes in glutamatergic neurons and glia cells in the cerebral cortex.

### Cell culture

PC12 cells were maintained and differentiated with NGF as previously described [18]. For NGF treatment, cells were grown in RPMI 1640 medium (Thermo Fisher Scientific) supplemented with 1% heat-inactivated horse serum (Sigma-Aldrich), penicillin/streptomycin (Thermo Fisher Scientific) and 50 ng/ml recombinant human β-NGF (Alomone Labs, Jerusalem, Israel) for 7-8 days in a 7,5% CO_2_ atmosphere at 37°C. Medium was changed every other day and before transfection.

HEK293 cells were cultured in DMEM medium supplemented with 10% fetal bovine serum and penicillin/streptomycin (all from Thermo Fisher Scientific) in a 5% CO_2_ atmosphere at 37°C.

Rat primary cortical cultures were prepared as previously reported [19]. Briefly, cortex from embryonic (E18) Sprague-Dawley rats were dissected out, dissociated and plated at a density of 250 cells/mm^2^ on poly-L-lysine-coated (Sigma-Aldrich) coverslips or at a density of 700 cells/mm^2^ on poly-L-lysine-coated plates. Neurons were maintained in neurobasal medium with B27 and 2mM GlutaMAX (all from Gibco). However, neurons plated on coverslips were initially seeded with MEM medium supplemented with 100 mM pyruvic acid (Gibco), 20% glucose (Sigma-Aldrich) and 10% heat-inactivated horse serum.

### Lentiviral preparation

Lentiviral particles were produced by transient transfection with Lipofectamine 2000 (Thermo Fisher Scientific) in HEK293 cells. 72 hours post-transfection, cell medium was collected, centrifuged to remove debris and filtered through 0.45 μm-pore filters (Thermo Scientific). Lentiviruses were concentrated by ultracentrifugation and resuspended in sterile PBS Ca2+/Mg2+ (Fisher Scientific). Viral titer was assessed by transduction of several viral dilutions.

### Transfection, treatments and viral infection

Primary rat cortical neurons, neuronal PC12 cells or HEK293 cells were transfected with Lipofectamine 2000 (Thermo Fisher Scientific) in accordance with the manufacturer's instructions. Neuronal PC12 cells transfection rate is around 15-20% and % of neuronal viral transduction is at least a 60%.

6-OHDA treatments were performed after 5-6 days of differentiation in NGF-treated PC12 cells. Medium was replaced right before exposure to the toxin.

Rat cortical neurons were infected at DIV 4 or at DIV 7 with lentiviral particles containing an ShRNA construct against rat NEDD4 (ShNEDD4) or a scrambled control sequence (ShCt), both purchased from Santa Cruz Biotechnologies. In the case of lentiviral particles containing ShCt and ShRTP801, cortical neurons were transduced at DIV 5. Lentiviral particles containing pHAGE, pHAGE-NEDD4 or pHAGE-NEDD4-C867S were infected at DIV 7. In all cases, neurons were infected at a multiplicity of infection (MOI) of 1.

### *In vitro* ubiquitination assay

The Ubiquitinylation Kit (Enzo Life Sciences) was used to perform the assay. Manufacturer's instructions were followed with minor modifications. Shortly, 1 mM dithiothreitol in 20 mM Tris-HCl pH 7.5, 20 U/ml inorganic pyrophosphatase, 5 mM Mg-ATP, 2,5 μM biotinylated ubiquitin, 0,1 μM His6-tagged recombinant human ubiquitin-activating enzyme E1, His6-tagged recombinant human ubiquitin-conjugating enzyme UbcH5b E2 (Enzo Life Sciences), recombinant human NEDD4 protein (Sigma Aldrich) and N-terminal GST-tagged recombinant human RTP801 full-length protein (Novus Biologicals, Littleton, CO, USA) were added to the reaction buffer, as specified in each condition, and then incubated at 37°C for 90 min. Next, RTP801 was immunoprecipitated using an anti-RTP801 antibody and equal volumes of each sample were analyzed by WB. Membranes were probed for biotinylated ubiquitin using Avidin/Biotin-Horseradish Peroxidase (Ultra-sensitive ABC staining kit; Thermo Fisher Scientific) or for RTP801.

### Western blot

Whole cell extracts and *NEDD4f/f;Emx1Cre* cortical lysates were collected and processed as previously described [6, 44]. Chemiluminiscent images were acquired using a LAS-3000 Imager (Fujifilm, Valhalla, NY, USA) and quantified by computer-assisted densitometric analysis (ImageJ).

### Immunoprecipitation and ubiquitination assay

Cell extracts were collected with RIPA buffer (50 mM Tris-HCl pH 7.4, 150 mM NaCl, 1% NP-40 (Sigma-Aldrich), 1% sodium deoxycholate (Sigma-Aldrich) and 0.1% sodium dodecyl sulfate (Sigma-Aldrich), containing mini cOmplete protease inhibitor (Roche Diagnostics Corporation, Indianapolis, IN, USA). Lysates were further solubilized by sonication and centrifuged for 10 min at 13000 g to remove insolubilities. Equal amounts of total protein were incubated overnight (O/N) on rotation at 4°C with protein A-agarose beads (Santa Cruz Biotechnology) and with the corresponding antibody or a normal immunoglobulin (Santa Cruz Biotechnology) as a negative control. Then the beads were centrifuged and washed four times with RIPA buffer and the immunocomplexes were collected and analyzed by WB.

### Co-immunoprecipitation

The cross-linking agent DSP (Thermo Fisher Scientific) was applied to cell cultures according to manufacturer's instructions. Then, cell extracts were collected with cell lysis buffer (Cell Signaling Technology). Equal amounts of total protein were incubated overnight (O/N) at 4°C on rotation with protein A-agarose beads (Santa Cruz Biotechnology) and with RTP801 antibody or a normal immunoglobulin (Santa Cruz Biotechnology). Beads were centrifuged, washed four times with CHAPS buffer (Tris 50 mM pH 7.4, NaCl 150 mM, MgCl_2_ 10 mM, CHAPS 0,4%), and the immunocomplexes were resolved in a WB.

### Quantitative reverse transcription-PCR

Total RNA was isolated from NGF-differentiated PC12 cells or rat primary cortical neurons with the High Pure RNA Isolation Kit (Roche Diagnostics Corporation, Indianapolis, IN, USA). cDNA reverse transcription from total RNA was performed by using the Transcriptor First Strand cDNA synthesis Kit (Roche Diagnostics Corporation). The following primers were used for quantitative PCR amplification: RTP801 forward primer, 5′-GCTCTGGACCCCAGTCTAGT-3′; RTP801 reverse primer, 5′-GGGACAGTCCTTCAGTCCTT-3′; NEDD4 forward primer, 5′- GGACGAGGTATGGGAGTTCT-3′; NEDD4 reverse primer, 5′- CTCCACTCATCGGGTCATAC-3′; α-actin forward primer, 5′-GGGTATGGGTCAGAAGGACT-3′; and α-actin reverse primer, 5′-GAGGCATACAGGGACAACAC-3′. Quantitative PCR was carried out with a 7500 Real Time PCR System (Applied Biosystems, Foster City, CA, USA) using equal amounts of cDNA template. Quantitative PCR analysis of RTP801 or NEDD4 was normalized by α-actin and analyzed using the comparative quantification.

### Immunofluorescence

NGF-differentiated PC12 cells and rat primary cortical neurons were fixed with 4% paraformaldehyde in PBS and stained as previously described [7]. Cortical neurons were permeabilized with PBS containing 0.25% Triton X-100 during 5 minutes, and blocked with superblock-PBS for 30 minutes. In cell survival assays, transfected viable cells were scored by strip counting [6].

### Immunohistochemistry of human sections

Postmortem human SNpc paraffin-embedded sections from sporadic PD patients and control individuals were obtained from the Neurological Tissue Bank (Biobank-HC-IDIBAPS) and stained as previously reported [6]. Sections were dewaxed with xylene and rehydrated by incubation in ethanol series (100%, 95%, 70% and 50%). Antigen retrieval was performed with Tris-EDTA buffer (10 mM Tris-base pH = 9, 1 mM EDTA, 0.05% Tween 20) in a vegetable steamer at 100°C for 20 minutes. Then, slides were blocked in superblock-PBS for 2 hours at room temperature and were incubated with avidin/biotin solution (Vector Laboratories, Burlingame, CA). Sections were incubated O/N at 4°C with anti-NEDD4 antibody (Abcam), washed with TBS-0.025% Triton X-100 and incubated for 15 minutes with 0.3% H_2_O_2_ in TBS. Secondary antibody incubation was performed for 2 hours at room temperature with biotinylated goat anti-rabbit secondary antibody (Vector Laboratories). Finally, slides were incubated with ABC Peroxidase Standard Staining Kit (Thermo Fisher Scientific) for 30 minutes and with ImmPACT SG Peroxidase Substrate (Vector Laboratories) for 20 minutes. Sections were washed, dehydrated by incubation in ethanol series (50%, 70%, 95% and 100%) and xylene, mounted with DPX mountant (Sigma-Aldrich) and examined under the microscope.

**Table 1:** Human brain samples information

PATIENT ID	CLINICAL DIAGNOSIS	ANATOMO-PATHOLOGICAL DIAGNOSIS	GENDER	AGE	TIME POSTMORTEM (hours)
CT1	Control	NFT II + alpha synuclein in olfactory bulb	Male	64	10
CT2	Control	AgD I	Male	83	13
CT3	Control	Cerebral metastasis from lung cancer + NFTI-II	Female	56	14
CT4	Control	Spine thrombosis D + cerebellar ictus +bulb, NFT III	Female	86	4
CT5	Control	iLBD Braak 1, NFT I-II, SVD	Male	78	6
CT6	Control	Minimum AgD I, with patched gliosis	Male	76	11,5
SPD1	PD	LBD Braak 4, ARP I B	Male	71	5
SPD2	PD	LBD Braak 4 + NFT Braak II	Male	77	12
SPD3	PD	LBD Braak 4, ARP II/A, discreet CAA, SVD	Male	88	15
SPD4	PD	LBD 4-5 + ARP IIA	Female	83	4
SPD5	PD	LBD Braak 5 + ARP III B + CAA mod	Male	74	8
SPD6	PD	LBD Braak 5 + AgD	Male	81	5

CT= control individuals, SPD= sporadic PD patients LBD: Lewy body disease, stages based on Braak et al. (2003): 1-6. ARP: Alzheimer-type pathology: classification of the neurofibrillary pathology NFT based on Braak et al., (2006) (I-VI) and the neuritic plaques based on CERAD criteria (A-C), CAA amyloid angiopathy. PMD: postmortem delay (in hours). AgD: Argyrophilic grain Disease, SVD Small Vessel Disease.

### Statistics

All experiments were performed at least in triplicate and results are expressed as the mean ± SEM. Statistical analyses were performed with unpaired Student's *t* test or when comparing multiple groups with one-way ANOVA with Bonferroni or Newman-Keuls multiple comparison test, as indicated in figure legends. Values of *P* < 0.05 were considered as statistically significant.

## Abbreviations

6-OHDA: 6-hydroxydopamine
DSP: dithiobis succinimidyl propionate
eGFP: enhanced green fluorescent protein
HMW: high molecular weight
IB: immunoblot
IP: immunoprecipitation
mTOR: mechanistic target of rapamycin
NEDD4: neural precursor cell expressed developmentally down-regulated protein 4
NGF: nerve growth factor
NM+: neuromelanin positive
PD: Parkinson's disease
shRNA: short hairpin RNA
SPD: sporadic Parkinson's disease
SNpc: substantia nigra pars compacta
UPS: ubiquitin proteasome system
WB: western immunoblotting
WT: wild-type
SNP: single nucleotide polymorphism.
